# Advancement of single-cell sequencing for clinical diagnosis and treatment of pancreatic cancer

**DOI:** 10.3389/fmed.2023.1213136

**Published:** 2023-08-31

**Authors:** Ke Zhang, Yuan Chen, Jie Zhu, Xinyu Ge, Junqing Wu, Peng Xu, Jie Yao

**Affiliations:** ^1^Dalian Medical University, Dalian, China; ^2^Medical College of Yangzhou University, Yangzhou, China; ^3^Northern Jiangsu People’s Hospital Clinical Medical College, Yangzhou University, Yangzhou, China

**Keywords:** single-cell sequencing, pancreatic cancer, heterogeneity, microenvironment, personalized therapy

## Abstract

Single-cell sequencing is a high-throughput technique that enables detection of genomic, transcriptomic, and epigenomic information at the individual cell level, offering significant advantages in detecting cellular heterogeneity, precise cell classification, and identifying rare subpopulations. The technique holds tremendous potential in improving the diagnosis and treatment of pancreatic cancer. Moreover, single-cell sequencing provides unique insights into the mechanisms of pancreatic cancer metastasis and cachexia, paving the way for developing novel preventive strategies. Overall, single-cell sequencing has immense potential in promoting early diagnosis, guiding personalized treatment, and preventing complications of pancreatic cancer. Emerging single-cell sequencing technologies will undoubtedly enhance our understanding of the complex biology of pancreatic cancer and pave the way for new directions in its clinical diagnosis and treatment.

## 1. Introduction

**Figure 1:**
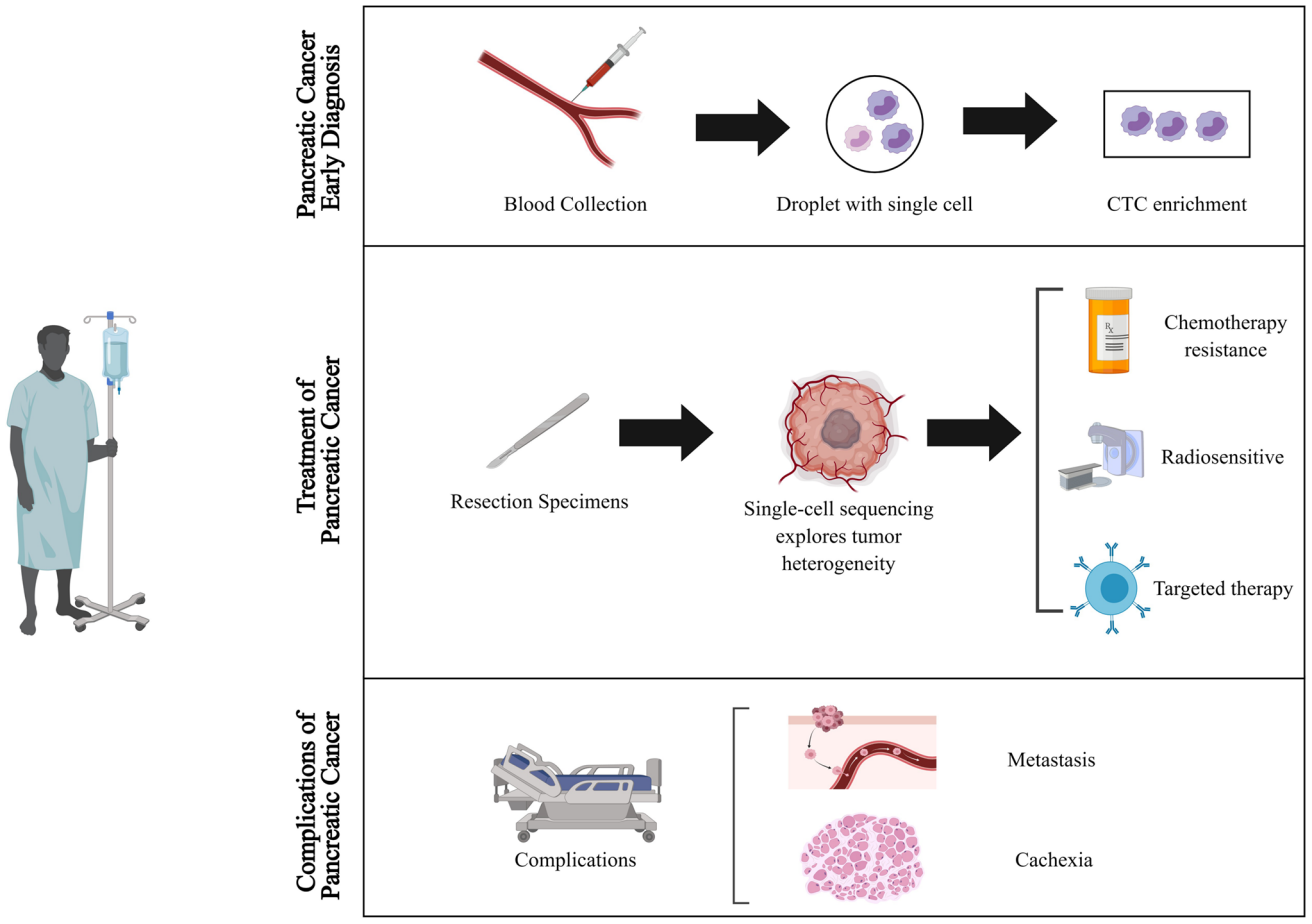
Applications of single-cell sequencing technology in the diagnosis and treatment of pancreatic cancer. Single-cell sequencing technology transforms the landscape of pancreatic cancer diagnosis and therapy. It unlocks novel dimensions by uncovering elusive cell populations and tumor heterogeneities for early detection, unraveling mechanisms of treatment resistance, tailoring therapies to individuals, and illuminating metastasis and cachexia pathways, thereby opening fresh avenues for preventive strategies. CTC, circulating tumor cells.

Pancreatic cancer is one of the deadliest cancers, with a 5 years survival rate of less than 10%. Globally, the three regions with the highest age-standardized incidence rates of pancreatic cancer are Western Europe (17.2 per100,000 person-years), Eastern Europe (15.5 per100,000 person-years), and North America (16.2 per100,000 person-years), followed by Australia (14.6 per100,000 person-years) and East Asia (11.8 per100,000 person-years) (1–3). Early diagnosis is crucial for improving the outcomes of pancreatic cancer, yet it remains a challenge due to the manifestation of non-specific symptoms primarily in the advanced stages of the disease. Currently, early diagnosis of pancreatic cancer relies on serum markers, such as CA 19-9 and CEA, as well as imaging techniques like CT and MRI. However, both methods suffer from low sensitivity and specificity, resulting in challenges for precise early detection (4–6). Surgical resection is currently the sole curative treatment for pancreatic cancer. Nevertheless, due to the advanced stage of the disease at the time of diagnosis, only a minority of patients are eligible for surgery. Chemotherapy and radiation therapy are the cornerstone of therapy for unresectable pancreatic cancer. Presently, the standard first-line treatment for advanced pancreatic cancer is gemcitabine-based chemotherapy. Combination chemotherapy regimens, such as FOLFIRINOX and nab-paclitaxel plus gemcitabine, have demonstrated improved survival rates in advanced pancreatic cancer (7, 8). Besides, recent efforts have focused on developing targeted therapies for pancreatic cancer, including drugs that target specific molecular alterations like the KRAS oncogene, which is frequently mutated in pancreatic cancer (9–11). Regardless of the therapeutic approach, the heterogeneity of pancreatic cancer cells and the complexity of its tumor microenvironment contribute to the emergence of drug resistance. Furthermore, patients with advanced pancreatic cancer commonly experience a range of complications including pain, biliary obstruction, cachexia, and venous thromboembolism (12). Cachexia, a chronic condition characterized by weight loss and muscle wasting, has a profound impact on the patient’s quality of life (13). Unfortunately, there are no universally agreed upon and effective preventive measures for this condition at present (14). Hence, continued advancements in diagnostic techniques, chemotherapy and radiation therapy regimens, targeted therapy strategies, and cachexia treatments will be critical for improving patient outcomes (Figure 1).

**Figure 2:**
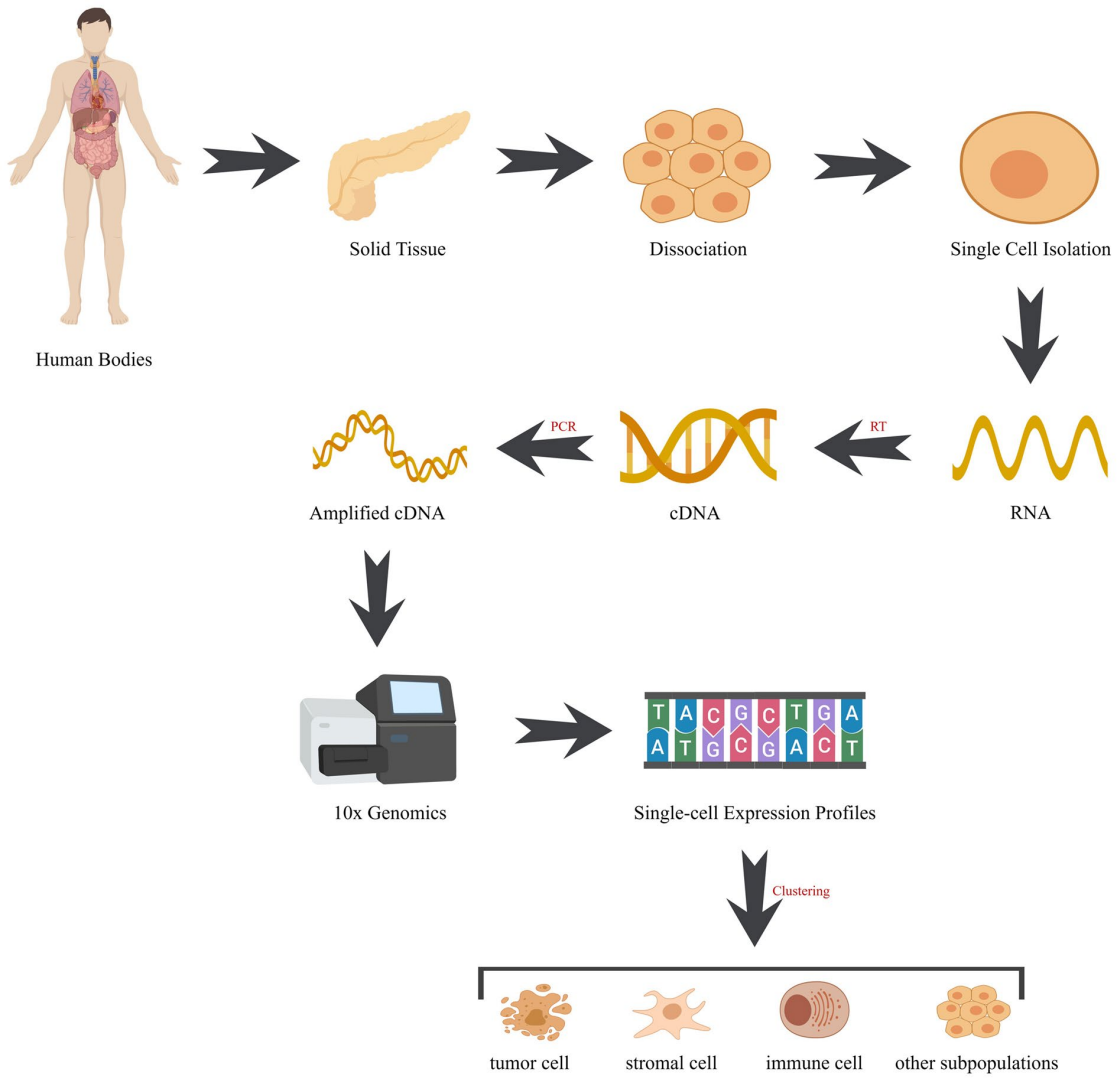
Workflow of single-cell sequencing. The workflow comprises pivotal stages: (1) cell isolation: employing techniques like microfluidics or fluorescence-activated cell sorting (FACS), cells are meticulously extracted from tissues. (2) RNA extraction: post-lysis, cellular RNA is meticulously captured, encompassing the entire transcriptome of RNA molecules. (3) Reverse transcription: RNA undergoes reverse transcription into complementary DNA (cDNA), acting as the sequencing template. (4) Library construction: fragmentation and adapter tagging of cDNA, followed by amplification, culminate in the formation of a sequencing library. (5) Sequencing: utilizing high-throughput sequencing, an array of succinct DNA sequences is generated from the library. (6) Data analysis: employing bioinformatics tools, sequences are aligned to a reference genome, enabling gene expression quantification and genetic variation identification. cDNA, complementary DNA; RT, reverse transcription; PCR, polymerase chain reaction.

The advent of single-cell sequencing technology has had a profound impact on our understanding of cellular heterogeneity and provided new insights into the complexities of human diseases. By analyzing individual cells, single-cell sequencing allows for the detection of rare or heterogeneous cell populations that would be otherwise obscured in bulk sequencing (15–17). With the aid of cutting-edge sequencing technologies like 10× genomics and drop-seq (Table 1), researchers can now conduct high-throughput analyses of thousands of single cells simultaneously, providing high-resolution profiling of the transcriptome, epigenome, and genome of individual cells (Figure 2) (21). Compared to bulk sequencing methods, single-cell sequencing has several advantages, including the ability to identify rare cell types or subpopulations and to detect genetic and epigenetic alterations at the single-cell level. Additionally, it offers valuable insights into the cellular heterogeneity and clonal evolution within complex tissues, and has the potential to discover new biomarkers and therapeutic targets for various diseases. Currently, single-cell sequencing has been broadly utilized in diverse fields of biological and medical research, such as developmental biology, immunology, microbiology, neurobiology, and particularly in cancer research (22–24). In the context of cancer, single-cell sequencing has provided insights into the genetic and epigenetic heterogeneity within tumors and their surrounding microenvironment, which has contributed to the discovery of novel therapeutic targets and treatment strategies (3, 25).

**Table 1:** Summary of the advantages and limitations of important single-cell sequencing technologies and platforms.

Technology	Platform	Advantages	Limitations	References
Droplet-based	10× genomics	1. High throughput: capable of covering a large number of cells 2. Potential for detecting rare cell types: due to its ability to analyze numerous cells 3. Distinct differential gene expression: captures unique characteristics of cell clusters	1. Higher noise for low-expressed mRNAs 2. Severe dropout issues: particularly evident for genes with lower expression levels 3. More non-coding genes detected: including a higher proportion of lncRNAs	(18)
Plate-based	Smart-seq2	1. Comprehensive gene detection: captures more genes per cell, especially low abundance and alternatively spliced transcripts 2. Resembles bulk RNA-seq data: composite data exhibits closer similarity to bulk RNA-seq profiles	1. Lower throughput: suitable for analyzing a limited number of cells 2. Higher proportion of mitochondrial genes captured 3. Less severe dropout problem: compared to the droplet-based platform, but with potentially limited coverage of rare cell types	(18)
Combinatorial indexing	CITE-seq/REAP-seq	1. Concurrent multi-dimensional revelations: adeptly extracts both cell transcriptome and surface protein information in parallel 2. Augmented data precision: communicates an all-encompassing amalgamation of cell phenotype and gene expression attributes	1. Stringent experimental requisites: mandates adept manipulation of experimental labeling and processing, with the potential to introduce technical variations 2. Elaborate data analysis: calls for sophisticated computational strategies to unravel the intricacies of integrated data interpretation	(19)
Tailored methodologies	In drop	1. Versatile open-source capability: offers the flexibility to embrace diverse chemistries and modifications, facilitating adaptation to diverse RNA-seq protocols 2. Economically efficient alternative: in drop presents a cost-effectiveness on par with 10×, with a per-cell expenditure roughly halved	1. Performance limitations: attributed to excessive cDNA amplification and incomplete protocol optimization 2. Reduced sensitivity: transcription detection sensitivity lower than other systems	(20)

CITE-seq, cellular indexing of transcriptomes and epitopes by sequencing; REAP-seq, RNA expression and protein sequencing assay; in drop, indexing drop RNA sequencing.

## 2. Applications of single-cell sequencing in pancreatic cancer early diagnosis

### 2.1. Single-cell sequencing for early diagnosis of pancreatic cancer

Pancreatic cancer is a highly malignant solid tumor with a poor prognosis for many patients, largely due to the lack of effective diagnostic methods and the fact that many cases are diagnosed at a late stage of the disease (26). Liquid biopsy is defined as the study of tumor biology from different blood sources. It has gained widespread adoption in the early diagnosis of solid tumors due to its advantages of simplicity and non-invasiveness, such as the detection of circulating tumor DNA (ctDNA) or circulating tumor cells (CTC) (27). Circulating tumor cells (CTCs) are shed from the tumor tissue and form distant metastases through circulation. Thus, as compared to ctDNA, CTCs are live tumor cells that may more accurately reflect the current tumor biology. In addition, the cultivation of CTCs *in vitro* allows for downstream functional assays and even personalized investigations, and CTCs have been demonstrated to hold prognostic and predictive value in pancreatic cancer (28–30). The accurate identification of circulating tumor cells (CTCs) has become a current research focus due to the limited number of CTCs in circulation and the difficulty of distinguishment from other cellular components in the bloodstream. Currently, common techniques for isolating CTCs include physical properties and surface markers. Separation based on physical properties, such as cell size and density, has the unique advantage of being unbiased and independent of cell surface markers, and enables differentiation between CTCs and blood cells. In contrast, cellular surface markers may only be present in a subset of CTCs due to their heterogeneity. Currently, there is no unified marker capable of identifying all CTCs while excluding all other cells in the blood (31). The most commonly used biomarker for identification and isolation of circulating tumor cells (CTCs) has been epithelial cell adhesion molecule (EPCAM). However, recent research indicates that EPCAM-negative CTCs also participate in the distant metastasis of cancer cells (32), indicating the need for alternative biomarkers for identification and isolation of CTCs.

Single-cell sequencing has enabled the analysis of heterogeneity in CTCs at the cellular level. Currently, several experiments have successfully developed novel surface markers by integrating single-cell sequencing data (33). In Zhu’s et al. study, they analyzed single-cell RNA sequencing profiles of pancreatic circulating tumor cells (CTCs) from genetically engineered mouse models (GEMMs) and pancreatic cancer patients. They found that compared to peripheral blood mononuclear cells (PBMCs), CLIC4 and GAS2L1 were overexpressed in pancreatic CTCs. In addition, using a combination of GAS2L1 and EPCAM expression increased the detection rate of pancreatic CTCs in GEMMs and patient samples (34). Therefore, the overexpression of GAS2L1 in pancreatic CTCs and its utility in combination with EPCAM as a cell surface marker represent a promising approach for detecting and isolating pancreatic CTCs, and highlights the potential of GAS2L1 as a novel biomarker for pancreatic cancer.

Recent studies have highlighted the crucial role of not only pancreatic CTCs but also stromal cells, particularly pancreatic stellate cells (PSCs), in the progression and metastasis of pancreatic cancer. In the microenvironment of pancreatic cancer, PSCs as resident cells play a critical role not only in the development and progression of pancreatic cancer but also in its metastasis. PSCs are characterized by their vitamin A-rich cytoplasmic lipid droplets in a quiescent state, whereas in the process of tumor progression, PSCs become activated and acquire a myofibroblast-like phenotype (35). A study was conducted to investigate the role of PSCs in metastasis and tumor angiogenesis. The experimental design involved injecting a mixture of male mouse PSCs and female mouse PC cells into the pancreas of female mice to evaluate the involvement of PSCs in distant metastasis of tumor cells. The study results revealed the presence of PSCs at multiple metastatic sites. Building on these findings, the team hypothesized the existence of circulating pancreatic stellate cells (cPSCs) and used single-cell sequencing to confirm their presence and characterize their gene expression levels. The study revealed that cPSCs exhibit increased expression of extracellular matrix protein-related genes, and that a low count of cPSCs was associated with a decreased risk and incidence of postoperative progression in pancreatic cancer (36). These findings suggest a potential role for cPSCs in the pathogenesis and progression of pancreatic cancer, and may have implications for the development of novel diagnostic and therapeutic approaches for this disease.

## 3. Single-cell sequencing and the treatment of pancreatic cancer

### 3.1. Single-cell sequencing and chemotherapy resistance

Besides the difficulty in early diagnosis leading to delayed optimal surgical timing, the resistance of pancreatic ductal adenocarcinoma (PDAC) to multiple drugs is also a major factor contributing to its poor prognosis. Although patients show high drug tolerance, all pancreatic cancer patients, regardless of whether they undergo surgical treatment, are subjected to broad-spectrum systemic chemotherapy (37). Currently, the first-line chemotherapy regimen used in clinical practice for PDAC is the FOLFIRINOX regimen (5-fluorouracil, leucovorin, irinotecan, and oxaliplatin), while the gemcitabine and nab-paclitaxel combination regimen is a second-line alternative. Although FOLFIRINOX regimen serves as an alternative option for gemcitabine-based chemotherapy, it only marginally prolonged overall survival of patients (38, 39), finding new treatment strategies to address gemcitabine resistance remains the most pressing issue at present. Although extensive research has been conducted to investigate the mechanism of gemcitabine resistance at the molecular level, such as epidermal growth factor receptor (EGFR), which has been identified to be associated with gemcitabine resistance in preclinical studies, a large-scale phase III clinical trial of gemcitabine in combination with selective EGFR inhibitor erlotinib did not significantly improve patient survival compared to gemcitabine monotherapy (40). This may be attributed to the high heterogeneity of PDAC and its complex tumor microenvironment. However, single-cell sequencing technology can analyze the heterogeneity of PDAC at the level of individual cells and depict the complex tumor microenvironment. Researchers utilized single-cell sequencing technology to screen pancreatic cancer cell lines to identify gemcitabine-resistant (GR) cells. Further differential gene analysis revealed upregulation of intracellular calcium signaling-related gene expression in GR cells, such as CALM2. Although GR cells exhibit an upregulation of intracellular calcium signaling-related genes, such as CALM2, single-cell RNA sequencing revealed that inhibition of calcium-dependent calmodulin activation resulted in the loss of the gemcitabine-resistant phenotype. This was due to impaired activation of the RAS/ERK signaling pathway. In subsequent *in vitro* cell experiments, depletion of extracellular calcium ions in the culture medium weakened ERK activation in GR cells and restored cellular sensitivity to gemcitabine treatment (41). Based on the results, the upregulated calcium signaling-related genes identified in GR cells selected through single-cell sequencing may serve as key mediators of gemcitabine resistance.

Previous studies have generally considered that chemotherapy mainly inhibits tumor cell proliferation by inducing DNA damage-mediated apoptosis. However, an increasing number of studies have found that chemotherapy drugs are also related to immune regulation in addition to directly killing tumor cells. A recent study has demonstrated that tumor cells undergoing chemotherapy-induced death release damage-associated molecular patterns (DAMPs), such as ATP and calreticulin, which can activate antigen-presenting cells (APCs) and stimulate T cells to ultimately generate a long-lasting antitumor immune response (42), which can activate antigen-presenting cells (APCs) and stimulate T cells to ultimately generate a long-lasting antitumor immune response. As a source of anti-tumor immune cells, it has been demonstrated through research that hematopoietic stem and progenitor cells (HSPCs) are enriched in the bone marrow of cancer patients, and ultimately differentiate into macrophages with immune function (43). Currently, the mechanism by which chemotherapeutic agents such as gemcitabine enhance tumor cell killing via immune modulation remains unclear. Vorontsova employed single-cell RNA sequencing to examine hematopoietic stem and progenitor cells (HSPCs) in mouse bone marrow in the context of PDAC. The results revealed that gemcitabine-treated mice showed a threefold increase in the number of megakaryocyte-erythroid progenitors (MEPs) compared to the control group. Furthermore, the transfer of MEPs to mice with pancreatic tumors substantially curbed tumor growth and elevated the levels of anti-tumor immune cells in both the peripheral blood and tumor microenvironment. Additionally, MEPs enhance the cytotoxic activity of CD8^+^ T cells and NK cells through the secretion of CCL5 and CXCL16 (44). Using single-cell sequencing to study HSPCs in mouse bone marrow, the researchers discovered that chemotherapy led to an increase in the concentration of MEPs and consequently bolstered the immune system’s ability to fight against pancreatic cancer. These findings highlight the promise of using MEPs as a therapeutic approach to augment the immune response to pancreatic cancer.

### 3.2. Single-cell sequencing and targeted therapy

#### 3.2.1. Precision immunotherapy: targeting the immune system to fight cancer

Although the primary treatment options for advanced PDAC currently remain systemic chemotherapy with gemcitabine or FOLFIRINOX, the existence of chemoresistance results in low survival rates for patients following chemotherapy regimens (45). Immunotherapy for cancer has shown significant efficacy in multiple malignancies. Currently, immunotherapy strategies are mainly divided into two categories based on whether the body produces adaptive immunity. Passive immunotherapy refers to the direct action of immune effector cells or molecules on tumor cells, including antibody-targeted therapy, adoptive immune cell therapy, and engineered T cell therapy. Active immunotherapy involves activating the adaptive immune process within the body to exert anti-tumor effects, with common treatment modalities including tumor vaccines (46–48). The discovery of programmed cell death protein 1 (PD-1) and programmed cell death ligand 1 (PD-L1) as targets for monoclonal antibodies has revolutionized the treatment of various malignant tumors, providing significant benefits to patients (48–50). Despite the identification of programmed cell death protein 1 (PD-1) and programmed cell death ligand 1 (PD-L1) as immune checkpoint inhibitor (ICPi) targets, the complex tumor microenvironment and high heterogeneity of PDAC tumor cells have hindered the efficacy of anti-PD-1/PD-L1 treatment. With the application of single-cell sequencing technology, it is now possible to investigate the underlying mechanisms of resistance to ICPi therapy at the cellular level, which may provide insights for the development of novel therapeutic strategies. A study using four orthotopic pancreatic cancer mouse models established by intrapancreatic injection of different pancreatic cancer cell lines showed that only the pancreatic tumors induced by pan02-h7 cells responded to anti-PD-1 therapy after a period of treatment. Further single-cell sequencing analysis of resistant and sensitive tumors revealed significant differences in infiltration of effector CD8^+^ T cells and tumor-associated macrophages (TAMs), with approximately 80% of TAMs expressing CD86 (an M1 marker) and less than 20% of TAMs expressing CD206 (an M2 marker) in the tumors induced by pan02-h7 cells (51). The single-cell sequencing results indicate that the imbalance in TAM differentiation may be a critical factor contributing to the suboptimal response to tumor immune checkpoint therapy.

Based on the aforementioned research findings, it is evident that the suboptimal response to immune checkpoint inhibitor therapy may be attributed to the immune-infiltrating cells present within the tumor microenvironment (TME). As the most abundant immune cells that infiltrate PDAC (52), TAMs can be categorized into two subgroups based on their different functions: immune-activating M1 macrophages and immune-regulating M2 macrophages. M1 macrophages activate anti-tumor immunity by secreting interferon-gamma (IFN-γ) and other inflammatory cytokines, while M2 macrophages produce immune-suppressive cytokines, such as interleukin-10 (IL-10), which participate in tumor immune evasion and promote tumor cell proliferation in the tumor microenvironment (TME) (53, 54). Thus, the development of therapeutic approaches targeting TAMs may offer a promising avenue to improve the unsatisfactory response to immune checkpoint inhibitor therapy. Although many studies have shown that targeting the CD47-SIRPα signaling pathway can inhibit tumor growth and prolong patient survival, the expression of CD47 in PDAC and its relationship with TAMs remain unclear (55, 56), despite the fact that signal regulatory protein α (SIRPα) as a transmembrane protein has been demonstrated to exist on macrophages and its ligand CD47 is widely present on the surface of tumor cells (57). Pan et al. explored the effect of CD47 blockade on TAMs and other immune-infiltrating cells using single-cell sequencing, presenting a novel therapeutic strategy. Their findings revealed that anti-CD47 therapy augmented pro-inflammatory TAMs and diminished anti-inflammatory TAMs, consequently reshaping the lymphocyte composition in mice with tumors. The investigators discovered that anti-CD47 therapy not only induced alterations in TAMs, but also increased the infiltration of intra-tumoral effector T cells and upregulated the expression of immune checkpoint receptors such as PD-1 in effector T cells. Moreover, combination therapy targeting CD47 and PD-L1 synergistically suppressed the growth of PDAC in a mouse model (58). Overall, single-cell sequencing technology has revealed the mechanism of TAM-targeted therapy, providing a theoretical basis for future combination therapy targeting TAMs and ICPi.

#### 3.2.2. Directing attention to the supporting cast: targeting the stroma

CAFs, which are a heterogeneous group of stromal cells and a crucial component of the TME (59), have been identified in pancreatic ductal adenocarcinoma (PDAC) through transcriptomic analysis in recent years (60, 61). Two main subtypes of CAFs have been classified in the TME based on their spatial distribution: myo-fibroblastic CAFs (myCAFs), which are located near tumor cells, and inflammatory CAFs (iCAFs), which are located away from tumor cells (62). Moreover, the use of single-cell sequencing technology has revealed additional subtypes of CAFs, including antigen-presenting CAFs (apCAFs) that co-express major histocompatibility complex class II (MHC II) and CD74 (63, 64). Research suggests that CAFs’ heterogeneity may be attributed to their origin from various cell types and activation through distinct signaling pathways (65, 66). Huang et al. integrated multiple PDAC single-cell sequencing datasets and found that apCAFs expressing MHC II mainly originate from mesothelial cells, which are induced by interleukin-1 (IL-1) and transforming growth factor β (TNF-β) to downregulate their mesothelial properties and acquire fibroblast properties during pancreatic cancer formation and differentiation (63). Many prior studies have highlighted the critical role of interactions between CAFs and other microenvironmental cells in tumor initiation and progression (67). A study using single-cell sequencing of pancreatic tumor tissue after KRAS inactivation found that, despite immune cell infiltration remaining unaffected, there was a reduction in the polarization of macrophages and other myeloid cells. Further investigations unveiled that CAFs were the principal source of cytokines that regulated macrophage polarization and were under the regulation of KRAS (68). The critical involvement of CAFs in extracellular signal transduction and the mediation of macrophage polarization is clearly evident.

The application of single-cell sequencing technology has yielded a more profound comprehension of the immune suppression response that is instigated by CAFs. This has resulted in a marked escalation of experiments that aim to restore anti-tumor immune responses by targeting CAFs in recent years. With regards to therapeutic strategies targeting cancer-associated fibroblasts (CAFs), the following are the main approaches at present: (1) depletion of CAFs directly by targeting surface markers; (2) inhibition of the activation and function of CAFs by targeting relevant effector molecules; (3) constraining the extracellular matrix (ECM) remodeling that is induced by CAFs. Previous research has demonstrated a strong correlation between the RAS/MEK/STAT3 pathway and resistance to treatment in PDAC (69). In the investigation conducted by Datta et al. (70), the use of MEK/STAT3 inhibitors (MEKi/STAT3i) in combination was found to diminish the number of pro-inflammatory and LRRC15^+^ myofibroblasts, and to enrich the population of CAFs expressing mesenchymal stem cell-like features, such as Ly6a/CD34, as revealed by single-cell sequencing analysis. These results suggest that the inhibition of relevant effector molecules could suppress the activation and function of CAFs, and may offer a promising new strategy for combating treatment resistance in PDAC. In a separate investigation, galectin-4 (Gal4), a protein secreted by tumor cells that accumulates in the ECM, was identified as a regulator of immune cell activity in PDAC. Single-cell sequencing analysis of a PDAC organoid model with Gal4 knocked down revealed an increase in the proportion of M1 macrophages, T cells, and antigen-presenting cells, which are known to benefit PDAC prognosis. Conversely, the proportion of M2 macrophages, which have immunosuppressive functions, was decreased. The results indicated that the reduced expression of Gal4 in the tumor immune microenvironment led to a widespread increase in anti-tumor activity, this encompassed an elevation in T cell activation, an upsurge in M1 macrophage polarization, a reduction in the proportion of immunosuppressive cells, and an increase in the proportion of antigen-presenting cells. After performing single-cell sequencing analysis on CAFs, it was discovered that myCAFs were more abundant in the Gal4 knockdown group, while iCAFs were less abundant compared to the control group. This implies that the decreased expression of Gal4 resulted in a reduction of inflammation. Thus, restricting CAF-induced ECM remodeling may be a potential strategy to improve the immunosuppressive microenvironment (71).

The Hedgehog signaling pathway is a critical regulatory pathway that governs the initiation, recurrence, growth, and metastasis of tumor cells. Numerous key nodes have been identified in the regulation of the Hedgehog pathway, including Hedgehog ligands, PTCH transmembrane receptors, SMO proteins, and glioma-associated oncogene (GLI) transcription factors, according to current research (72). While the Hedgehog pathway plays a critical role in regulating pancreatic tumor cells, the reported role of the Hedgehog signaling pathway in the occurrence and progression of pancreatic cancer has been inconsistent. In the research conducted by Olive et al. (73), the administration of IPI-926, a SMO inhibitor, in a PDAC mouse model was found to significantly reduce the formation of ECM and improve the therapeutic effectiveness of gemcitabine. However, subsequent clinical trials have yielded completely opposite results. Vismodegib, an SMO inhibitor approved by the FDA for the treatment of advanced basal cell carcinoma, was found by researchers to not improve the therapeutic efficacy of gemcitabine and did not improve the survival of PDAC mice in a combination therapy of vismodegib and gemcitabine (74). In a phase II clinical trial (NCT01088815), 67 untreated patients with metastatic PDAC were evaluated, and the results indicated that the combination therapy with vismodegib did not improve overall survival of the patients compared to historical data of chemotherapy alone (75). The substantial discrepancy observed in the aforementioned results could be attributed to the high heterogeneity of both tumor cells and the microenvironment. Therefore, elucidating the impact of the Hedgehog signaling pathway on tumor cells and the microenvironment at the cellular level becomes especially critical. Using single-cell sequencing technology, Steele and colleagues investigated the expression of components of the Hedgehog signaling pathway in different cell types of both normal pancreatic tissue and PDAC. The study findings demonstrated that myCAFs exhibited higher Hedgehog pathway activation levels compared to iCAFs in PDAC. Furthermore, inhibiting the Hedgehog signaling pathway altered the proportion of myCAFs and iCAFs in PDAC, leading to the enrichment of iCAFs that promote tumor growth (76). In summary, while the combination of Hedgehog signaling pathway targeting and standard chemotherapy is ineffective for PDAC, the use of single-cell sequencing technology has highlighted the close relationship between specific signals and other components in the tumor microenvironment. This paves the way for the future development of innovative therapeutic approaches targeting the Hedgehog signaling pathway.

### 3.3. Shining a light on radiosensitivity: the power of single-cell sequencing

As previously noted, although conventional first-line chemotherapy regimens and emerging targeted therapies in recent years have demonstrated some degree of resistance in clinical treatment of PDAC, this has ultimately resulted in unsatisfactory therapeutic outcomes. With the advancement of minimally invasive techniques for treating tumors, a growing number of local ablation methods have been employed in cancer treatment. Among these methods, radiofrequency ablation (RFA) has emerged as the most frequently employed approach for locally ablating tumors in clinical practice (77). As an effective local treatment method for a variety of solid tumors, RFA induces coagulative necrosis of local tumor tissue, which releases tumor antigens and subsequently stimulates the host’s adaptive immune response against the tumor (78). A recent study has demonstrated that RFA treatment not only triggers a localized immune response at the treatment site, but also generates an effective antitumor immune response against non-RFA tumors in distant locations. Fei et al. utilized single-cell sequencing technology to assess the immune cell infiltration in non-RFA treated tumors within a mouse model of pancreatic ductal adenocarcinoma. They observed an augmented proportion of functional T cells in non-RFA tumors at distant sites. Additionally, the team discovered that RFA treatment modified the gene expression profile of diverse cell clusters at the single-cell level. For instance, after RFA treatment, immune checkpoint genes (PD-1 and LAG3) were upregulated in T cells located in non-RFA tumors in distant locations (79). The single-cell sequencing results have confirmed that local RFA treatment can remodel the immune microenvironment of non-RFA tumors in distant locations, and the combination of RFA with immune checkpoint inhibitors has potential as an effective treatment. Despite being the most prevalent minimally invasive treatment for tumors, RFA still faces some challenges due to its reliance on the thermal conduction strategy for killing tumor cells, which can lead to major blood vessel damage and the heat sink effect. Compared to RFA, nanosecond pulsed electric field (nsPEF) is able to effectively prevent damage to adjacent organs caused by thermal energy (80). nsPEF has demonstrated its efficacy in eliminating tumor cells in mouse pancreatic cancer, and subsequent investigation utilizing single-cell sequencing technology has unveiled that the local ablation response induced by nsPEF can promote immune stimulation. For instance, researchers have discovered that while the tumor volume of mouse models receiving nsPEF therapy decreased, there was an increase in the infiltration of macrophages and dendritic cells that participate in the formation of immunosuppressive tumor microenvironments (81). It is evident that the utilization of nsPEF for ablative therapy in pancreatic cancer is effective. However, further refinement of treatment protocols is necessary to prevent potential immune suppressive reactions during the course of treatment.

## 4. Single-cell sequencing and complications of pancreatic cancer

### 4.1. Unraveling metastasis mysteries with single-cell sequencing

Pancreatic cancer is a malignancy with high invasiveness, causing worsening of patients’ conditions and reduction of their survival rates due to complex complications. Metastasis, which is one of the most prevalent complications of pancreatic cancer, is characterized by the spread of malignant cells through the bloodstream or lymphatic system to other organs, including the liver, lungs, peritoneum, bones, and brain. Hence, an in-depth understanding of the mechanism by which pancreatic cancer spreads to distant sites is imperative for mitigating its metastatic potential and ameliorating the prognosis of affected patients. Presently, investigations into the mechanism of pancreatic cancer metastasis predominantly center around epithelial-to-mesenchymal transition (EMT), which describes the acquisition of mesenchymal characteristics by epithelial cells (82, 83). Numerous studies have revealed that EMT is not simply a one-step transformation, but a complex and continuous process governed by multiple factors that is closely linked to tumor metastasis and resistance to treatment (82, 84, 85). Leveraging the power of single-cell sequencing technology, a comprehensive and detailed analysis of cell lineages involved in the continuous process of EMT can be attained with precision and accuracy. In a study on liver metastasis of PDAC, Carstens and colleagues identified that the cell lineage of EMT is highly heterogeneous and the EMT program is highly plastic, through single-cell sequencing of human and mouse PDAC. They further that inhibiting the EMT program can enable tumor cells to acquire a stable epithelial phenotype and help tumor cells adapt to liver metastasis settlement (86). The results indicate that the utilization of single-cell sequencing can unveil the intricate mechanisms that control EMT, thus laying the groundwork for the development of novel therapeutic strategies to fight against cancer metastasis and improve patient prognosis.

### 4.2. Shedding light on cachexia with single-cell sequencing

Cachexia, in addition to metastasis, is another significant complication of advanced pancreatic cancer. Cachexia is a wasting syndrome that often accompanies various chronic diseases, characterized by involuntary weight loss, muscle wasting, abnormal lipid metabolism, anorexia, fatigue, and an inability to be completely reversed by traditional nutritional support, ultimately leading to progressive organ dysfunction (87). As one of the malignancies with the poorest prognosis, pancreatic cancer has the highest incidence of cachexia among all types of malignant tumors (88, 89). Multiple factors contribute to the aforementioned phenomenon, including metabolic changes associated with pancreatic cancer tumor biology, disruptions in pancreatic digestion and endocrine function, and the distinctive anatomical location of the pancreas (90). In recent years, there has been increasing interest in studying metabolic changes in pancreatic cancer. Researchers have noted that pancreatic cancer cells often have KARS mutations and upregulation of glycolytic enzymes in mutant tumor cells, enabling them to continually metabolize glucose to adapt to the nutritional deficiencies caused by cachexia. Furthermore, researchers have elucidated that tumor cells can surmount metabolic stress elicited by the adverse tumor microenvironment through alternative pathways, including ramping up macropinocytosis to catabolize proteins and sustain the adaptive capacity of tumor cells in environments deprived of adequate nutrition (91). During single-cell sequencing analysis of normal pancreatic tissue, early-stage, and late-stage pancreatic cancer, genes linked to macropinocytosis were found to increase in expression as the disease progressed. Among these genes is acyl-CoA synthetase short-chain family member 2 (ACSS2). A closer examination of ACSS2’s downstream genes revealed that it enhances macropinocytosis through the downstream ETV4/ZIP4 pathway, while ZIP4 promotes cachexia via the GSK3βB/TRAIL axis, ultimately resulting in tumor cachexia (92). Therefore, it is clear that ACSS2 supports the development of tumor cells by mediating macropinocytosis and muscle wasting. The development of drugs targeting ACSS2 is expected to delay the progression of cachexia in pancreatic cancer.

Furthermore, in addition to the metabolic alterations in the tumor cells themselves, stromal cells within the tumor microenvironment of pancreatic cancer also undergo metabolic changes during tumor progression (93). With the development and application of single-cell sequencing, researchers have acquired a more profound comprehension of the specific mechanisms involved in the metabolic interaction between tumors and the stroma. Previous research has revealed that, as the major amino acid liberated during muscle catabolism, glutamine (Gln) plays a pivotal role as a carbon source in the tricarboxylic acid (TCA) cycle of pancreatic cancer cells (94, 95). In Liu’s et al. study, single-cell sequencing technology was used to analyze the expression of Gln metabolic enzymes in stromal cells and tumor cells. The results showed that compared to tumor cells, stromal cells exhibited higher levels of glutamine synthetase (GS). Further knockdown experiments revealed that the loss of GS disrupted Gln synthetic metabolism in stromal cells, especially PSCs, and ultimately led to the inhibition of pancreatic cancer cell growth (96). The results of the above study suggest that, abnormal metabolism in stromal cells is also intimately linked to tumor growth. Besides metabolic alterations in tumor cells and their microenvironment, external behavioral factors like anorexia promote the development and advancement of cachexia. Owing to its unique anatomical location, the hypothalamus assumes a pivotal role in the regulation of diverse behavioral responses involved in cachexia. In previous studies, the medial basal hypothalamus (MBH) has been identified as one of the driving factors of metabolic dysregulation in cancer cachexia, but the underlying molecular mechanisms remain unclear (97–99). Multiple cell types in the MBH are affected by tumor-derived factors that are induced by tumor growth, leading to a marked change in the microenvironment of neurons critical for behavioral, metabolic, and neuroendocrine outputs dysregulated during cachexia. For instance, investigators have discovered that the increased expression of lipocalin-2 (LCN2), a lipid-binding protein, in the MBH, impacts the gene expression of POMC, a neuron linked to feeding behavior. This implies that molecules identified through single-cell sequencing could serve as promising therapeutic targets for mitigating cachexia symptoms (100).

## 5. Conclusion

The advent of single-cell sequencing technology has transformed the landscape of cancer research, including pancreatic cancer. It facilitates the characterization of gene expression, mutations, and epigenetic modifications at a single-cell resolution, offering an in-depth comprehension of the tumor’s cellular heterogeneity. Nevertheless, this technology has its limitations, and several hurdles still need to be overcome to apply it effectively in clinical research for pancreatic cancer. One major limitation of single-cell sequencing technology is the throughput of current single-cell sequencing platforms is limited, making it difficult to analyze large numbers of cells in a timely and cost-effective manner (3). Moreover, the reliability and reproducibility of single-cell sequencing data can be affected by various factors, such as technical variability and sample quality. Another challenge is the complexity and heterogeneity of pancreatic cancer, which requires the identification of multiple subpopulations of tumor cells and non-cancerous cells in the tumor microenvironment. In addition, the lack of standardization in single-cell sequencing protocols and data analysis methods makes it difficult to compare results across different studies and platforms (3, 101, 102).

To overcome these challenges, several strategies have been proposed. One approach is to integrate single-cell sequencing data with other omics data, such as bulk RNA sequencing, DNA sequencing, and proteomics, to provide a more comprehensive understanding of the molecular landscape of pancreatic cancer. Another strategy is to improve the accuracy and reproducibility of single-cell sequencing data through the development of standardized protocols and quality control measures. Additionally, the development of more advanced single-cell sequencing platforms, can increase the throughput and reduce the cost of single-cell sequencing.

In conclusion, while single-cell sequencing technology has significantly advanced our understanding of the cellular heterogeneity of pancreatic cancer, several challenges remain. The development of more cost-effective and high-throughput single-cell sequencing platforms, as well as standardization of protocols and data analysis methods, will be critical for the future application of this technology to clinical research in pancreatic cancer.

## Author contributions

KZ, YC, JZ, XG, JW, PX, and JY contributed to the study conception and design and commented on previous versions of the manuscript. The first draft of the manuscript was written by KZ. All authors contributed to the article and approved the submitted version.

## Conflict of interest

The authors declare that the research was conducted in the absence of any commercial or financial relationships that could be construed as a potential conflict of interest.

## Publisher’s note

All claims expressed in this article are solely those of the authors and do not necessarily represent those of their affiliated organizations, or those of the publisher, the editors and the reviewers. Any product that may be evaluated in this article, or claim that may be made by its manufacturer, is not guaranteed or endorsed by the publisher.

## Figures and Tables

**Table:** 

PDAC	pancreatic ductal adenocarcinoma
TME	tumor microenvironment
GR	gemcitabine-resistant
EMT	epithelial-to-mesenchymal transition
ASR	age-standardized incidence rates
CA19-9	carbohydrate antigen 19-9
CEA	carcinoembryonic antigen
FOLFIRINOX	folinic acid (leucovorin), fluorouracil, irinotecan, and oxaliplatin
KRAS	Kirsten rat sarcoma viral oncogene homolog
EGFR	epidermal growth factor receptor
DAMPs	damage-associated molecular patterns
ICPi	immune checkpoint inhibitor
PD-1	programmed cell death protein 1
PD-L1	programmed cell death ligand 1
MHC II	major histocompatibility complex class II
RFA	radiofrequency ablation
nsPEF	nanosecond pulsed electric field
FACS	fluorescence-activated cell sorting
cDNA	complementary DNA
RT	reverse transcription
PCR	polymerase chain reaction
CITE-seq	cellular indexing of transcriptomes and epitopes by sequencing
REAP-seq	RNA expression and protein sequencing assay
in drop	indexing drop RNA sequencing
ECM	extracellular matrix
EPCAM	epithelial cell adhesion molecule
CTC	circulating tumor cells
GEMMs	engineered mouse models
PBMCs	peripheral blood mononuclear cells
PSCs	pancreatic stellate cells
APCs	antigen-presenting cells
HSPCs	hematopoietic stem and progenitor cells
CAFs	cancer-associated fibroblasts
myCAFs	myo-fibroblastic cancer-associated fibroblasts
iCAFs	inflammatory cancer-associated fibroblasts
apCAFs	antigen-presenting cancer-associated fibroblasts
TAMs	tumor-associated macrophages
TCA	tricarboxylic acid cycle
